# Plasticity of Performance Curves Can Buffer Reaction Rates from Body Temperature Variation in Active Endotherms

**DOI:** 10.3389/fphys.2017.00575

**Published:** 2017-08-04

**Authors:** Frank Seebacher, Alexander G. Little

**Affiliations:** ^1^School of Life and Environmental Sciences, University of Sydney Sydney, NSW, Australia; ^2^Rosenstiel School of Marine and Atmospheric Science, The University of Miami Miami, FL, United States

**Keywords:** thermoregulation, body temperature, climate, metabolism, mitochondria, AMPK, thyroid hormone, transient receptor potential ion channel

## Abstract

Endotherms regulate their core body temperature by adjusting metabolic heat production and insulation. Endothermic body temperatures are therefore relatively stable compared to external temperatures. The thermal sensitivity of biochemical reaction rates is thought to have co-evolved with body temperature regulation so that optimal reaction rates occur at the regulated body temperature. However, recent data show that core body temperatures even of non-torpid endotherms fluctuate considerably. Additionally, peripheral temperatures can be considerably lower and more variable than core body temperatures. Here we discuss whether published data support the hypothesis that thermal performance curves of physiological reaction rates are plastic so that performance is maintained despite variable body temperatures within active (non-torpid) endotherms, and we explore mechanisms that confer plasticity. There is evidence that thermal performance curves in tissues that experience thermal fluctuations can be plastic, although this question remains relatively unexplored for endotherms. Mechanisms that alter thermal responses locally at the tissue level include transient potential receptor ion channels (TRPV and TRPM) and the AMP-activated protein kinase (AMPK) both of which can influence metabolism and energy expenditure. Additionally, the thermal sensitivity of processes that cause post-transcriptional RNA degradation can promote the relative expression of cold-responsive genes. Endotherms can respond to environmental fluctuations similarly to ectotherms, and thermal plasticity complements core body temperature regulation to increase whole-organism performance. Thermal plasticity is ancestral to endothermic thermoregulation, but it has not lost its selective advantage so that modern endotherms are a physiological composite of ancestral ectothermic and derived endothermic traits.

## Introduction

**Figure 1:**
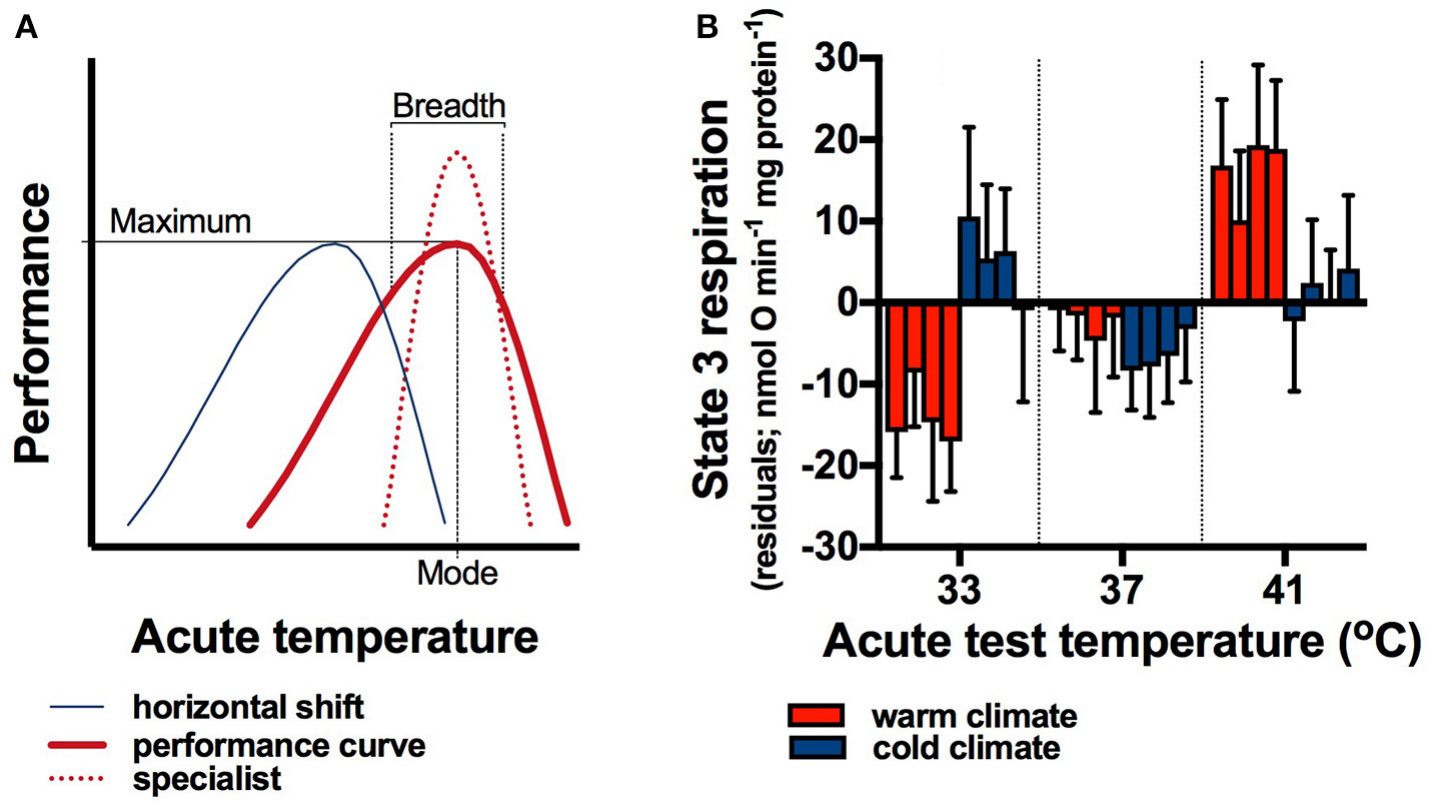
Responses of animals to variable environments. Thermal performance curves (**A**; thick red line) have a maximum at the optimal temperature (mode), and decreasing performance at either side of the maximum. The performance breadth, typically defined as the temperature range over which performance is greater than 80–90%, can change in response to temperature variation, producing specialist phenotypes (broken red line) with a narrower performance breadth but greater maximum. Plastic responses to temperature variation as a result of developmental processes or reversible acclimation can shift the performance curve so that the mode coincides with a different mean temperature (blue line), which may be advantageous for endotherms that experience lower body temperatures in colder climates. Thermal performance curves of maximal mitochondrial respiration rates (state 3 rates) shifted between populations of bush rats (*Rattus fuscipes*) living in different climates **(B)**. Rats from cold climate populations had significantly lower body temperatures than those from warm climate populations (Glanville et al., 2012). Concomitantly to body temperature differences, state 3 respiration rate was highest at low temperatures in cold climate rats, but it increased with increasing temperature in warm climate rats (climate^*^test temperature interaction), indicating that thermal performance curves shifted to compensate for the lower body temperatures in cold climates. Residuals are shown here, and within each group of four bars within acute test temperatures the first (left) bar shows data from vastus lateralis muscle, the second from heart ventricle, the third from liver, and the forth (right) bar shows data from brown adipose tissue. Means ± s.e.m. are shown, *n* = 10 rats from each population (averaged within climates), and data measured at different temperatures are separated by a thin dotted line to aid in visual clarity.

The basic principles of thermodynamics dictate that the rates of physiological functions in both endotherms and ecotherms are sensitive to changes in temperature (Landeira-Fernandez et al., 2012; Tattersall et al., 2012; Arcus et al., 2016; Else, 2016). However, the relationship between temperature and reaction rates is not constant (Huey and Kingsolver, 1989; Kingsolver, 2003). Thermal performance curves represent the change in a physiological reaction rate across a range of acute temperatures (Figure 1A). Thermal plasticity in response to a (non-acute) chronic change in body temperature may manifest as a horizontal shift in the performance curve, so that maximal performance (mode) occurs at the new temperature. Additionally, the range of values around the mode within which performance remains high (e.g., >80% of maximal) can increase or decrease, leading to generalist and specialist phenotypes, respectively (Figure 1; Huey and Kingsolver, 1989; Sinclair et al., 2016). The resultant plasticity of reaction rates is advantageous because it permits animals to maintain relatively constant physiological rates in variable environments (Guderley, 1990; St-Pierre et al., 1998; Piersma and Drent, 2003; Forsman, 2014). Ectothermic animals, in particular, benefit from such phenotypic plasticity, because body temperatures are largely determined by environmental conditions (Porter and Gates, 1969).

In endotherms, the gradient between body temperature and environmental temperature impacts metabolic rates and heat production, which typically increase under cold conditions (Rezende et al., 2004; Lovegrove, 2005; McKechnie et al., 2015). Importantly, body temperatures of non-torpid endotherms are not constant (Boyles et al., 2013; Hetem et al., 2016; Levesque et al., 2016). By lowering body temperatures in cooler environments, for example, even active (non-torpid and non-hibernating) endotherms reduce the differential between internal and external temperatures and can thereby reduce the energy needed for thermoregulation (Crompton et al., 1978; Glanville et al., 2012; Tattersall et al., 2016), resulting in increased survival and fitness (Dammhahn et al., 2017). Any changes in body temperature, however, will negatively affect cellular reaction rates unless these are buffered by plastic responses similar to those described above, which are common among ectotherms (Huey and Kingsolver, 1989).

Similarly, body temperatures are not homogenous within organisms, and even in non-dormant endotherms temperatures in peripheral muscle are often several degrees lower than core body temperature (Mutungi and Ranatunga, 1998; Yaicharoen et al., 2012). Again, reduced peripheral body temperatures lower the energetic costs of thermoregulation, but need to be accompanied by shifts in thermal performance curves of peripheral tissues to avoid a trade-off between thermoregulatory cost and physiological function.

Here we suggest that plasticity of thermal performance curves is an ancestral trait that has been maintained in endotherms to buffer physiological reaction rates from variation in core body or tissue temperatures. Note that there is an important distinction between acclimation of metabolic rates to increase heat production in response to cold environmental temperatures (e.g., Boratyński et al., 2017; Noakes et al., 2017), and the plasticity of performance curves we are suggesting (Figure 1A). The former serves to maintain body temperatures in variable climates, and the latter optimizes reaction rates when tissue temperatures change despite adjustments of metabolic heat production. Here we review plasticity in performance curves in response to core body temperature variation, and in response to peripheral tissue temperature variation in non-torpid and non-hibernating endotherms. Additionally, we review mechanisms that can confer plasticity in thermal performance curves at the tissue level, which are promising candidates for future research aimed at understanding the consequences of heterothermy in endotherms.

## Plasticity of performance curves in response to core body temperature variation

Physiological rates in endotherms tend to be optimized around the regulated core body temperature with relatively narrow performance breadth (Shinoda et al., 1997; James, 2013). However, there are exceptions to this pattern. Round-tailed ground squirrels (*Spermophilus terreticaudus*), for example, let their body temperature vary considerably with environmental temperature (Wooden, 2004). At the same time, their muscle force production and sprint speed was maintained over body temperatures ranging from 30 to 41°C. These data exemplify an extreme generalist phenotype, where the benefits of variation in body temperature are not traded off for a decrease in performance.

In addition to generalist responses, the mode of thermal performance curves may shift in response to body temperature changes. Heart rate represents a physiological rate that is closely related to performance (Eliason et al., 2011; Hillman and Hedrick, 2015), and thermal performance curves of heart rate can shift in response to chronic changes in body temperature. For example, mean rectal temperature of humans significantly decreased following cool acclimation, but was elevated to 39°C following acclimation to hot conditions (Racinais et al., 2017). Initially, heart rates were higher in hot conditions but decreased following acclimation to hot temperatures, indicating a shift in thermal sensitivity of heart rates (Racinais et al., 2017). On the other hand, red deer (*Cervus elaphus*) and Przewalski's horse (*Equus ferus przewalskii*) lowered peripheral temperatures in winter, with concomitant decreases in heart rates during activity and rest (Arnold et al., 2004, 2006). However, there was no indication that heart rates were compensated for the lower winter temperatures.

In a rodent (the Australian bush rat, *Rattus fuscipes*), physiological reaction rates shifted with seasonal and altitudinal changes in climate. *R. fuscipes* from two populations living in cold high altitude climates had significantly lower body temperatures compared to those from two warm coastal populations (Glanville et al., 2012). Paralleling differences in body temperatures, the thermal sensitivity of mitochondrial respiration rates differed significantly between populations from different climates. We published mitochondrial substrate oxidation rates (state 3 rates) and uncoupled (state 4) oxygen consumption rates measured at 37°C test temperatures previously (Glanville et al., 2012). However, at the same time (and using the same techniques as in Glanville et al., 2012) we also measured mitochondrial respiration at 33° and 41°C, and these previously unpublished data provide an opportunity to compare thermal sensitivities between populations experiencing different body temperatures naturally. Hence, here we used a permutational analysis (Wheeler and Torchiano, 2016) to analyse state 3 mitochondrial substrate oxidation rates (data for state 4 rates available from the corresponding author) with climate and test temperature as independent factors, and population nested within climate. We calculated residuals for each population to analyse thermal sensitivity without the effects of differences in absolute rates between populations (see Glanville et al., 2012).

In rats from warm climates, residuals of state 3 rates were lowest at 33°C and increased with temperature. In contrast, state 3 rates of cold-climate rats were highest at 33°C and decreased with increasing temperature (Figure 1B; climate^*^test temperature interaction, *p* < 0.0001 for muscle, *p* = 0.04 for heart, *p* = 0.008 for liver, and *p* < 0.0001 for brown adipose tissue; there was no effect of population on any residuals, all *p* > 0.12). These data indicate that adaptation or developmental processes lower the mode of thermal performance curves in cold climates, which would be beneficial for rats experiencing lower body temperatures.

Similarly, rats had significantly lower body temperatures in winter compared to summer (Glanville and Seebacher, 2010a,b). There were interactions between season and test temperature in determining metabolic enzyme activities, which indicate that the mode of performance curves shifted in response to seasonally changing temperatures as well (Glanville and Seebacher, 2010b).

## Plasticity of performance curves in response to tissue temperature variation

Even when core body temperature remains stable within organisms, there can be considerable temperature variation in peripheral tissues (Ponganis et al., 2003). Muscle temperatures in humans may be several degrees Celsius below rectal or core body temperatures at rest, even in large muscle groups (Sargeant, 1987; Ducharme et al., 1991; Bishop, 2003). These decreases in temperature constrain muscle performance (James, 2013), leading to increased sporting performance following warm-ups (Bishop, 2003; Yaicharoen et al., 2012; Cunniffe et al., 2017).

It would be advantageous therefore if the thermal sensitivity of performance curves differed between core and peripheral tissues. Muscle at the core should show greater thermal sensitivity with a narrow performance breadth around core body temperature, while peripheral muscle should be less sensitive to temperature changes and perform better than core muscle at low temperatures. Such a division between generalist and specialist phenotypes was found in isolated mouse muscle (James et al., 2015). Core diaphragm muscle had greater power output at core body temperature and was more sensitive to changes in temperature than peripheral soleus muscle, which would experience much greater temperature fluctuations *in vivo* (James et al., 2015). These patterns of regional specialization were similar in an endothermic shark that maintains elevated core body temperatures (Bernal et al., 2005). Endotherms can therefore show regional specialization correlated with temperature variation.

In mouse muscle, the capacity to shift performance curves in response to temperature is independent from central neuroendocrine input and can occur in isolated cells (Little and Seebacher, 2016). Cool (32°C) growth temperature of muscle precursor cells (myoblasts) lowered the mode of the thermal performance curve for metabolic rate compared to control (37°C) conditions, where increased metabolic rate at 32°C compensated for the negative thermodynamic effects. Similarly, decreased differentiation temperature (32°C) of myoblasts into functional myotubes lowered the mode of the thermal performance curve for metabolic rate, again compensating for cool temperatures. Interestingly, myoblast growth temperature influenced myotube thermal performance independently from differentiation temperature (Little and Seebacher, 2016). These thermal responses of mouse myocytes are comparable to the interaction between developmental and reversible plasticity in ectotherms (Scott and Johnston, 2012; Little et al., 2013; Beaman et al., 2016).

## Mechanisms mediating plasticity

Plasticity of thermal performance curves may be regulated centrally via sympathetic output from the hypothalamus (Nakamura and Morrison, 2007; Seebacher, 2009), and peripherally by circulating levels of thyroid hormone (Little and Seebacher, 2013, 2014; Little et al., 2013). However, the capacity to adjust thermal sensitivity in response to local changes in temperature can also occur independently from neuroendocrine input (Al-Fageeh et al., 2006; Underhill and Smales, 2007; Ye et al., 2013; Little and Seebacher, 2016). For example, changes in membrane fluidity resulting from temperature-sensitive shifts in fatty acid profiles can maintain cellular, organelle, and protein function in variable thermal environments (Cossins and Prosser, 1978). Changes in membrane composition occur at the cellular level in ectotherms and endotherms (Dymond, 2016; Ballweg and Ernst, 2017).

**Figure 2:**
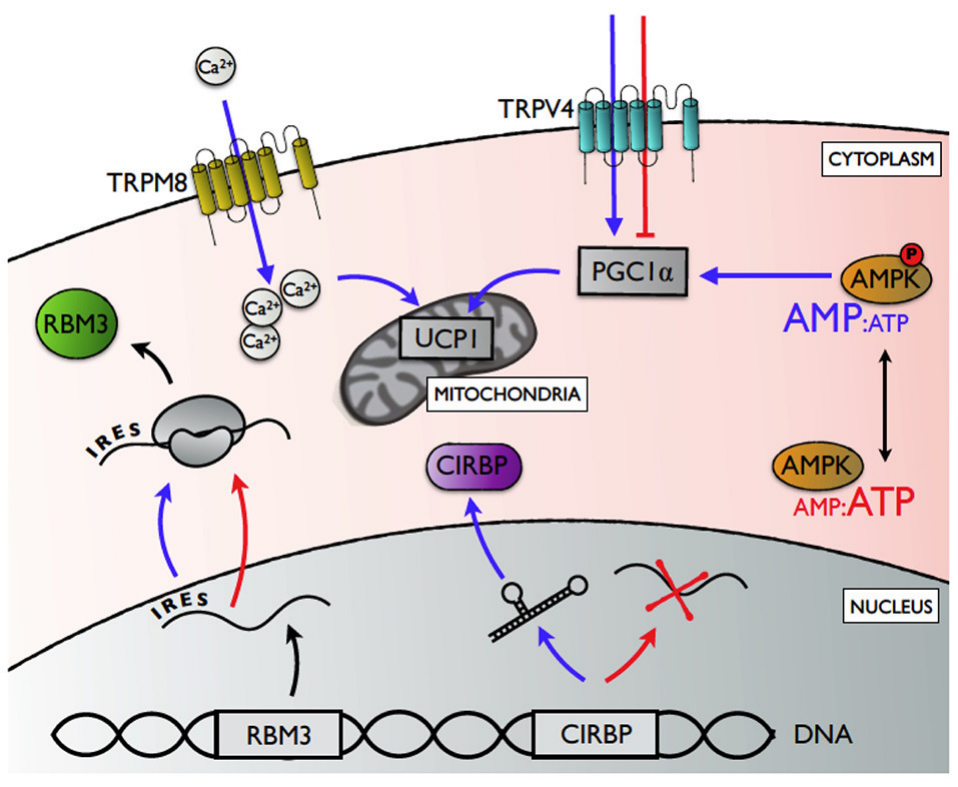
A summary of the potential mechanisms underlying cell-autonomous thermal plasticity. Transient receptor potential channels vanilloid 4 (TRPV4) and melastatin 8 (TRPM8) upregulate expression of peroxisome proliferator-activated receptor γ coactivator 1α (PGC1α) and uncoupling protein 1 (UCP1), respectively, in response to local hypothermia (blue arrows; normothermic conditions depicted by red arrows). Cold-inducible RNA binding protein (CIRBP) expression is enhanced in response to cold exposure through a temperature-sensitive change in RNA splicing that determines the proportion pre-mRNA processed into mature mRNA. Relative translation rates for RNA binding motif protein 3 (RBM3) is enhanced during cold exposure via a 5' internal ribosome entry site (IRES), while global protein synthesis declines. AMP-activated protein kinase (AMPK) is activated by increasing ratio of AMP:ATP with cold exposure, thereby enhancing PGC1α activity.

There has been considerable medical interest in cell-autonomous pathways regulating thermal responses in mammalian tissues (Ye et al., 2013; Borowiec et al., 2016; Quesada-López et al., 2016; Bastide et al., 2017). Additionally, thermal responses have been used as a biotechnology strategy to enhance recombinant protein production in mammalian cell lines (Al-Fageeh et al., 2006). Together these studies point toward a local “thermal switch” in peripheral tissues, where thermosensory information is integrated with compensatory response(s) entirely at the level of the cell. The “thermal switch” may comprise pathways that sense temperature changes directly, and those that sense subsequent imbalances in important cellular metabolites following thermal change (Figure 2).

Transient receptor potential ion channels (TRPs) represent the best studied mechanism that allows cells to detect changes in their environment directly (Nilius and Voets, 2005; Ahern, 2013). Many of these receptors are temperature-gated, and different receptors are activated at specific temperature ranges (Baez et al., 2014). TRPs are expressed ubiquitously, and are best known for their afferent role in thermoregulation (Caterina, 2006), where peripheral changes in temperature are relayed to the preoptic area of the hypothalamus for central regulation (Morrison et al., 2014). Interestingly, certain TRPs can also regulate local responses in a cell-autonomous manner (Ahern, 2013; Ye et al., 2013). In mammals, TRP vanilloid 4 (TRPV4) is activated at physiological temperatures (Shibasaki et al., 2015). TRPV4 knockout mice showed increased energy expenditure in white adipocytes (Ye et al., 2012), mediated by the cell-autonomous release of TRPV4-induced repression of the metabolic co-regulator PGC1α, and uncoupling protein 1 (UCP1) (Ye et al., 2012). TRP melastatin 8 (TRPM8), which detects cool temperatures (<26°C) (Bautista et al., 2007), can regulate cell-autonomous responses to hypothermia in brown adipose tissue (BAT) and germ cells of mice (Ma et al., 2012; Borowiec et al., 2016). In cold-exposed BAT, for instance, TRPM8 enhanced Ca^2+^-influx and increased the expression of UCP1 independently from the canonical β-adrenergic pathway (Ma et al., 2012). Our results, showing that mild hypothermia (32°C) altered metabolic phenotypes of myoblasts and subsequent myotubes (Little and Seebacher, 2016), are especially interesting because brown adipocytes and skeletal myocytes both share a myf-5 positive mesenchymal stem cell origin (Seale et al., 2008), which means that TRPM8 activation may also underlie metabolic programming to compensate for mild hypothermia in skeletal muscle development, repair, and maintenance.

Temperature can also have direct effects on post-transcriptional processes, such as mRNA degradation, splicing, and translation efficiency. For example, temperature-dependent expression patterns of cold-inducible proteins are determined by the thermal sensitivity of post-transcriptional mechanisms (Sonna et al., 2002; Potla et al., 2015; Gotic et al., 2016; Bastide et al., 2017). The expression of the cold-inducible cold shock RNA-binding protein (CIRBP) increased in response to mild hypothermia (Sonna et al., 2002), UV irradiation (Yang and Carrier, 2001), and hypoxia (Wellmann, 2004) in mammals. CIRBP was also upregulated during cold exposure in ectothermic vertebrates, including common carp (*Cyprinus carpio*; Gracey et al., 2004) and Japanese treefrogs (*Hyla japonica*; Sugimoto and Jiang, 2008). CIRBP regulated cell growth at low temperature by protecting target mRNA from degradation in mammalian cell culture (Phadtare et al., 1999). Increases in cellular CIRBP content in response to body temperature variation occurred through a temperature-sensitive change in RNA splicing efficiency that determined the proportion of CIRBP pre-mRNA processed into mature mRNAs (Gotic et al., 2016). Pre-mRNA contain secondary structures that regulate splice site recognition and splicesome binding. These secondary structures are dynamic and highly sensitive to changes in temperature, so that they can destabilize and unfold in ways that determine ultimate levels of CIRBP mRNA expression (Gotic et al., 2016).

The cold-shock RNA-binding protein RBM3 binds mRNAs to maintain translational efficiency (Peretti et al., 2015; Zhu et al., 2016; Bastide et al., 2017). Cooling reduces protein synthesis globally, except for specific proteins such as RBM3 (Bastide et al., 2017). The translation of RBM3 is enhanced during cold exposure by internal ribosome entry site (IRES) regions in its mRNA 5′ untranslated regions (5-UTR) (Bastide et al., 2017). Under cold exposure, the typical cap-dependent initiation of translation is impaired (Jackson et al., 2015; Bastide et al., 2017). However, IRES regions recruit the translational machinery, thereby facilitating initiation of translation in a cap-independent manner (Chappell et al., 2001; Pan and van Breukelen, 2011). As a result, RBM3 expression is maintained, or even enhanced with cold exposure. In addition to facilitating translational efficiency of mRNA in the cold, RBM3 can also regulate microRNAs by facilitating their processing by Dicer (Zhu et al., 2016). Cold exposure increased transcript levels for five microRNAs involved in cell cycle progression in primary cultured human small airway epithelial cells (Potla et al., 2015). These microRNAs are regulated post-transcriptionally, but it is not known whether their changes in expression rely on stabilization by RBM3, or temperature-sensitive splicing mechanisms similar to CIRBP (Potla et al., 2015).

Peripheral cells and tissues may also mount autonomous responses to local changes in temperature by indirect thermosensory pathways, where temperature-induced imbalances in cellular metabolites trigger compensatory responses. In skeletal muscle, decreasing temperatures cause an energy deficit, resulting in an increase in the AMP:ATP ratio (Towler and Hardie, 2007). Increased concentrations of AMP activate the cellular energy-sensor AMP-stimulated protein kinase (AMPK) to increase mitochondrial density and ATP production (Jäger et al., 2007; Lira et al., 2010; Hardie et al., 2016). Increases in AMPK activity shifted mouse muscle to a more oxidative metabolic phenotype (Ljubicic et al., 2011), and altered the expression of thyroid receptors in adipose tissue (Wang et al., 2014). The AMPK-mediated response can thereby alter how cells and tissues respond to central inputs via changes in receptor profiles, as well as acting on the metabolic machinery at the cellular level directly. As a result, metabolic capacity is increased in response to cold. AMPK may thereby represent a thermal switch by integrating temperature-induced energy deficit with compensatory cellular responses.

## Conclusions

The concept that endotherms have high and stable body temperatures despite environmental temperature fluctuations (Scholander et al., 1950; Rezende and Bacigalupe, 2015) has been challenged by the increasing evidence that body and tissue temperatures of non-torpid and non-hibernating endotherms can fluctuate substantially (e.g., Hetem et al., 2016; Levesque et al., 2016). Consequently, the notion that optimal physiological reaction rates of endotherms have evolved to be fixed within a narrow range of regulated body temperatures is also questionable. Instead, body and tissue temperature fluctuations in endotherms would favor selection for thermal plasticity. Endothermic thermoregulation is distinct from that of ectotherms, but thermal plasticity of physiological reaction rates can be as advantageous in endotherms as in ectotherms.

The mechanisms that mediate thermal plasticity are highly conserved among animals, and their broad range of functions is likely to preclude negative selection. For example, thyroid hormone action is essential for a broad range of physiological responses in animals, and is highly conserved across taxa (Heyland and Moroz, 2005; Darras and Van Herck, 2012). It is likely that thyroid hormone has retained its early functions as well as assuming additional roles in endotherms (Cannon and Nedergaard, 2010; Little, 2016). Similarly, AMPK-mediated signaling evolved in early eukaryotes as an energy sensing mechanism (Towler and Hardie, 2007; Hardie et al., 2016; Ross et al., 2016). Hence, the role of AMPK in conferring thermal plasticity evolved in ectothermic organisms and has been retained by endotherms. In a final example, transient receptor potential ion channels (TRP) act in thermoregulation in both ectotherms and endotherms (Caterina, 2006; Seebacher and Murray, 2007; Laursen et al., 2016). Like thyroid hormone and AMKP, TRPs are highly conserved among animals (Peng et al., 2015). These three mechanisms, and possibly others, such as micro RNAs and post-transcriptional modifications, therefore represent evolutionarily conserved regulatory systems that adjust cellular responses to the environment.

## Author contributions

FS and AL conceived the ideas, prepared the manuscript and figures, and approved the manuscript.

### Conflict of interest statement

The authors declare that the research was conducted in the absence of any commercial or financial relationships that could be construed as a potential conflict of interest.
